# Supplementary material for: Cortical neuroprosthesis-mediated functional ipsilateral control of locomotion in rats with spinal cord hemisection
Source: eLife. 2024 Nov 25;12:RP92940. doi: 10.7554/eLife.92940 (PMC11588340; doi:10.7554/eLife.92940)
Supplement: Supplementary file 1. — Rats marked with * did not receive left motor cortex implantation. They were included in the study for establishing spontaneous changes in posture over time (Figure 5A and B). [file elife-92940-supp1.docx]

|  |  |  |  |  |  |  |  |  |  |  |  |
| --- | --- | --- | --- | --- | --- | --- | --- | --- | --- | --- | --- |
| Rat # | **Intact rats (Fig. 1)** | **SCI Rats (Fig. 3)** | **EMG Analysis (Fig. 4)** | **Long-term efficacy (Figure 3-Figure supplement 3)** | **Posture : SCI severity (Fig. 5)** | **Posture : Recovery (Fig. 5)** | **Motor maps (Fig. 6)** | **Ipsilateral modulation (Fig. 7)** | **Contra ablation (Fig. 8)** | **Long-train stimulation (Fig. 9)** | **Injury severity group** |
| 1 | **√** | **√** | **√** | **√** | **√** | **√** | **√** |  |  |  | **Moderate** |
| 2 | **√** | **√** | **√** | **√** | **√** | **√** | **√** |  |  |  | **Severe** |
| 3 | **√** |  |  |  | **√** | **√** | **√** |  |  | **√** | **Moderate** |
| 4 | **√** | **√** | **√** |  | **√** |  | **Array failure** |  |  |  | **Moderate** |
| 5 | **√** | **√** | **√** |  | **√** | **√** | **√** | **√** |  | **√** | **Mild** |
| 6 | **√** | **√** | **√** |  | **√** | **√** | **√** |  |  | **√** | **Severe** |
| 7 |  | **√** | **√** |  | **√** | **√** | **√** |  |  |  | **Mild** |
| 8 |  | **√** | **√** |  | **√** | **√** | **√** | **√** |  | **√** | **Moderate** |
| 9 |  |  |  |  | **√** | **√** | **Array failure** |  |  | **√** | **Moderate** |
| 10 |  |  |  |  | **√** | **√** | **√** |  |  | **√** | **Moderate** |
| 11 |  |  |  |  | **√** | **√** | **√** |  |  |  | **Severe** |
| 12 |  |  |  |  | **√** | **√** | **√** |  |  |  | **Moderate** |
| 13 |  |  |  |  | **√** | **√** | **√** |  |  |  | **Mild** |
| 14 |  |  |  |  | **√** | **√** | **Array failure** |  |  |  | **Severe** |
| 15 |  |  |  |  | **√** | **√** | **Array failure** |  |  |  | **Moderate** |
| 16 |  |  |  |  | **√** | **√** | **√** |  |  |  | **Severe** |
| 17 |  |  |  |  |  |  |  |  |  | **Ketamine** |  |
| 18 |  |  |  | **√** |  |  |  |  |  | **Ketamine** |  |
| 19 |  |  |  | **√** |  |  |  |  |  | **Ketamine** |  |
| 20 |  |  |  | **√** |  |  |  |  |  | **Ketamine** |  |
| 21 |  |  |  |  | **√** |  |  |  | **√** |  |  |
| 22 |  |  |  |  | **√** |  |  |  | **√** |  |  |
| 23 |  |  |  |  | **√** |  |  |  | **√** |  |  |
| 24* |  |  |  |  | **√** |  |  |  |  |  | **Severe** |
| 25* |  |  |  |  | **√** |  |  |  |  |  | **Moderate** |
| 26* |  |  |  |  | **√** |  |  |  |  |  | **Moderate** |
| 27* |  |  |  |  | **√** |  |  |  |  |  | **Mild** |
| 28* |  |  |  |  | **√** |  |  |  |  |  | **Moderate** |
| 29* |  |  |  |  | **√** |  |  |  |  |  | **Moderate** |
| 30* |  |  |  |  | **√** | **√** |  |  |  |  | **Moderate** |
| 31* |  |  |  |  | **√** | **√** |  |  |  |  | **Mild** |
| 32* |  |  |  |  | **√** | **√** |  |  |  |  | **Moderate** |
| 33* |  |  |  |  | **√** | **√** |  |  |  |  | **Moderate** |
| 34* |  |  |  |  | **√** | **√** |  |  |  |  | **Moderate** |
| 35* |  |  |  |  | **√** | **√** |  |  |  |  | **Moderate** |
| 36* |  |  |  |  | **√** | **√** |  |  |  |  | **Mild** |
| 37* |  |  |  |  | **√** | **√** |  |  |  |  | **Moderate** |
| 38* |  |  |  |  | **√** | **√** |  |  |  |  | **Severe** |
| 39* |  |  |  |  | **√** | **√** |  |  |  |  | **Mild** |
| 40* |  |  |  |  | **√** | **√** |  |  |  |  | **Moderate** |
| 41* |  |  |  |  | **√** | **√** |  |  |  |  | **Moderate** |
| 42* |  |  |  |  | **√** | **√** |  |  |  |  | **Severe** |
| 43* |  |  |  |  | **√** | **√** |  |  |  |  | **Moderate** |
| 44* |  |  |  |  | **√** | **√** |  |  |  |  | **Moderate** |
| 45* |  |  |  |  | **√** | **√** |  |  |  |  | **Moderate** |
| 46* |  |  |  |  | **√** | **√** |  |  |  |  | **Moderate** |
| 47* |  |  |  |  | **√** | **√** |  |  |  |  | **Mild** |
| 48* |  |  |  |  | **√** | **√** |  |  |  |  | **Moderate** |
